# Detection of mandibular condylar fractures on head CT scans in infants with witnessed falls

**DOI:** 10.1007/s11282-026-00930-w

**Published:** 2026-05-08

**Authors:** Bethelhem Belachew, Marcus Meneses, Taisa Guarilha, Carmen R. Cerron-Vela, Karuna Shekdar, Savvas Andronikou

**Affiliations:** Department of Radiology, Children’s Hospital of Philadelphia, 3401 Civic Center Blvd, Philadelphia, PA 19104 USA

**Keywords:** Mandibular fractures, Non-accidental trauma, Pediatric facial trauma, Infant

## Abstract

Infants experience facial fractures infrequently due to their limited mobility, and when such injuries do occur, the mandible is among the most affected facial bones. Isolated mandibular condylar fractures, however, are rarely reported in this age group. Clinical detection is particularly challenging in nonverbal infants, making careful imaging assessment essential when evaluating facial trauma. This case series presents infants with condylar fractures that were first identified on head CT studies, emphasizing key clinical considerations and the importance of evaluating the mandibular condyles on every head CT.

## Introduction

Pediatric facial fractures are uncommon in children under five years of age. Their low incidence has been attributed to several factors, including a smaller face-to-cranium ratio and increased adipose cushioning in younger children [1]. Among these rare fractures, mandibular fractures represent 20–50%, and the incidence of condylar fractures is reported to be higher in children compared to adults [2, 3].

The mandibular condyle develops primarily through endochondral ossification at secondary growth centers, with Meckel’s cartilage acting only as a transient scaffold [4]. The condylar cartilage exhibits high biological activity during infancy and childhood, making it particularly susceptible to trauma [1, 2, 4]. Consequently, condylar fractures in pediatric patients, if unrecognized or inadequately managed, may interfere with normal mandibular growth and temporomandibular joint development [1, 2, 5].

Mandibular condyle fractures in children are often missed at first clinical presentation. Kannari et al. found that 14.8% of pediatric mandibular fractures were missed at first healthcare contact [4]. The only clinical finding significantly associated with missed fractures was the presence of a skin wound on the jaw [4], which underscores the need for careful systematic examination of mandibular condyles in all pediatric facial trauma, as condylar fractures are almost always associated with chin injuries [5]. Undiagnosed or improperly managed condylar fractures can result in disabling long-term sequelae, including temporomandibular joint ankylosis, facial asymmetry and deformities, malocclusion, and chronic pain [2, 6].

Imaging is essential for accurate diagnosis and characterization of pediatric mandibular condyle fractures [7]. Age-related anatomical variations in condylar size, shape, and angulation can mimic fractures, particularly in children. Recognition of these developmental and anatomical variants is therefore essential to distinguish normal features from true fractures and to guide appropriate clinical management [1, 2, 5, 8]. Because infants lack the ability to report symptoms and often exhibit subtle or no physical findings, radiological identification of mandibular condylar fractures is critical in the evaluation and management of these patients. Some of these cases with condylar fractures may also need further evaluation with a skeletal survey in cases of incongruent history or additional injuries. We aimed to highlight key radiologic features and practical considerations for diagnosis and management of condylar mandible fractures in a series of 5 infants with condylar fractures incidentally identified on imaging.

## Case presentation

### Case 1

**Fig. 1:**
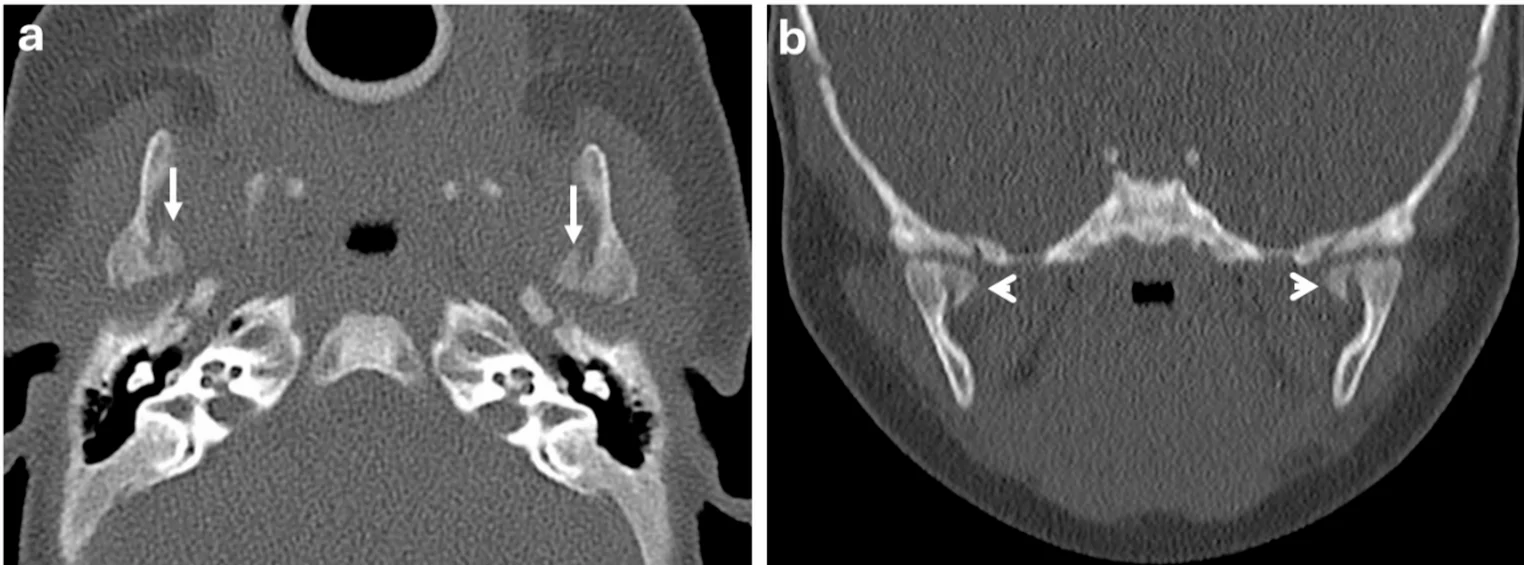
CT imaging (bone window) of a 4-week-old infant. **a** Axial image and **b** Coronal reconstruction demonstrate bilateral fractures running vertically through the mandibular condyles (*arrows*). The fracture lines extend into the Temporo-Mandibular Joint articular surfaces bilaterally, associated with minimal downward displacement of the medial fracture fragments (*arrowheads*)

A 4-week-old female presented to the emergency department after falling face-first from her 4-year-old cousin’s arms. She cried immediately and had no loss of consciousness. She was breastfeeding after the fall and prior to arrival to ED. Physical exam was unremarkable. Head CT was done for indication of head trauma using automated tube current to minimize radiation achieving mean volumetric CT dose index (CTDL vol) of 22.35 mGy and a total dose-length product (DLP) of 308 mGy-cm. Findings revealed unexpected bilateral nondisplaced mandibular condyle fractures, but no intracranial hemorrhage or skull fracture. Further evaluation with a face CT with similarly low dose protocols confirmed no additional mandibular or facial fractures (Fig. 1). The history was consistent with accidental trauma, and Non-Accidental Trauma (NAT) concern was low. The oral-maxillofacial surgeon recommended conservative management and follow-up after 2 weeks. The patient missed her scheduled follow-up and returned two months later for evaluation of a rash. During this visit, bruising was noted on the left lower extremity. The mother’s explanation of the injury was inconsistent with the age of the contusion. Given the repeat injury within three months and the discrepancy in history, the patient was evaluated for possible NAT - a skeletal survey was normal.

### Case 2

**Fig. 2:**
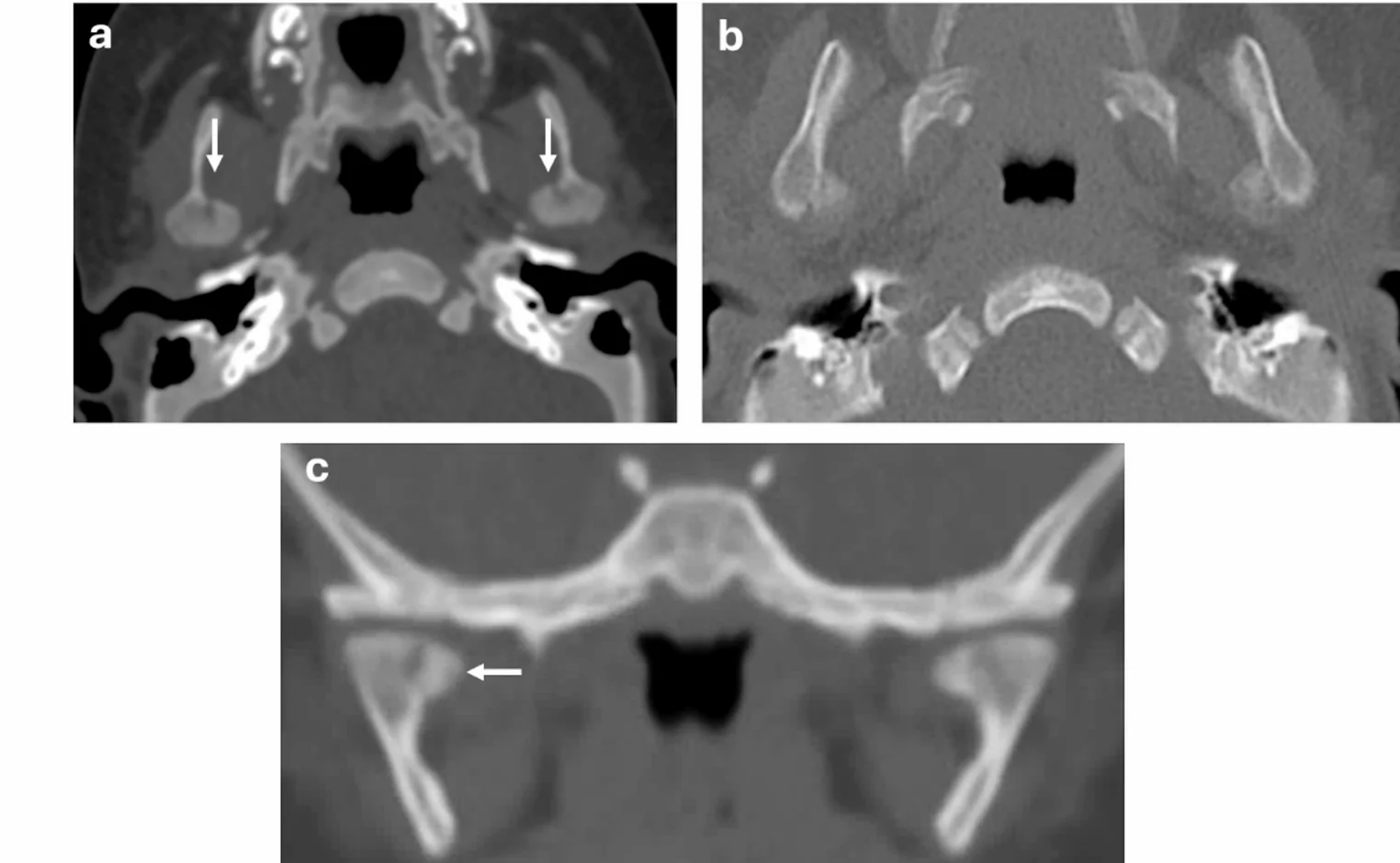
CT imaging (bone window) of a 3-month-old infant after a fall from a highchair. **a**, **b** Axial slices and **c** Coronal reconstruction demonstrate bilateral fractures of the mandibular condyles (*arrows*) extending into the joint space. There is no significant displacement of the fractured segments

A 3-month-old female presented after a witnessed fall from a highchair (approximately 4 ft) onto a hardwood floor while under parental supervision. She cried immediately, had no loss of consciousness, and physical examination was unremarkable. Head CT with low dose protocol (CTDL vol – 17.8 mGy and total DLP of 244.15 mGy-cm) revealed multifocal subarachnoid hemorrhage, no calvarial fractures but bilateral nondisplaced mandibular condyle fractures (Fig. 2). Given these findings, the patient underwent NAT evaluation, including physical and ophthalmologic exams and a skeletal survey, all of which were normal. The provided history was then considered an acceptable and developmentally plausible mechanism, and the injuries identified were considered as accidental trauma. The oral-maxillofacial surgeons did not recommend surgical intervention and booked follow-up for after 2 weeks.

### Case 3

**Fig. 3:**
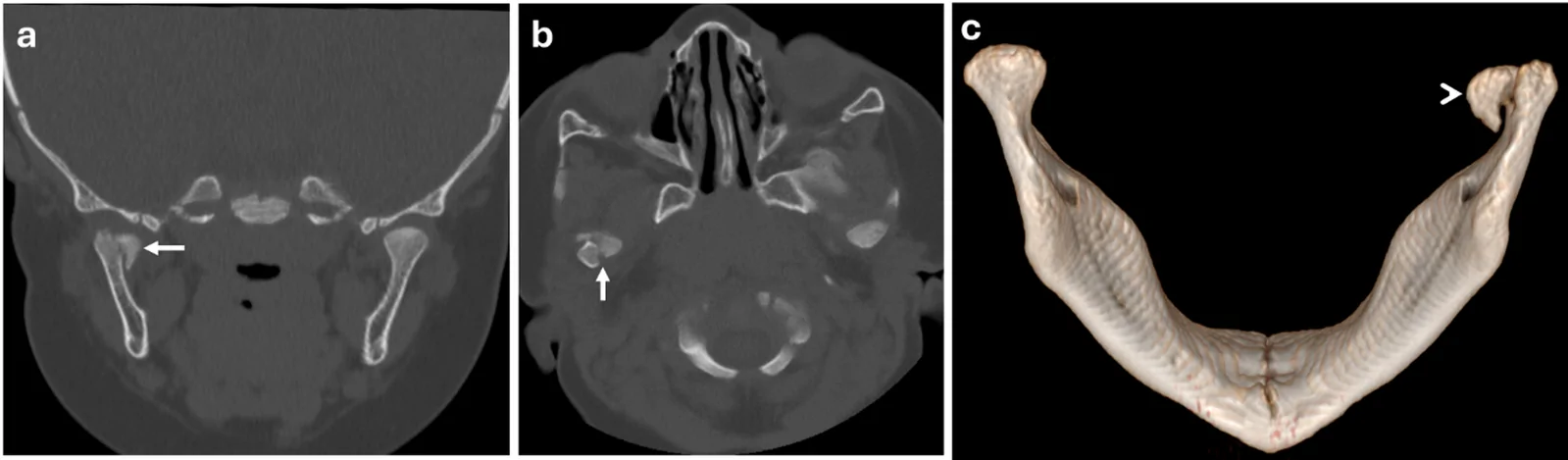
CT scan (bone window) of a 6-month-old girl following a fall from a kitchen table. **a** Coronal and **b** axial images show a comminuted, displaced intracapsular fracture of the right mandibular condyle (*arrow*), extending into the temporomandibular joint articular surface with inferomedial displacement of the medial fragment. **c** Posterior view of 3D reconstruction demonstrates the displaced medial fragment (*arrowhead*)

A 6‑month‑old female infant presented after a reported fall from a kitchen table. The mother stated that she found the infant prone on the floor, crying immediately. Following the event, the child demonstrated feeding refusal and avoided any oral contact, including pacifiers and teething toys. Physical examination, including inspection of the oropharynx and facial structures, showed no obvious external trauma. Initial non-contrast CT of the head revealed a right-sided, minimally displaced mandibular condylar fracture extending to the articular surface. Subsequent maxillofacial CT done with dose optimization for age and weight (CTDL vol – 25.12 mGy and total DLP of 319.95 mGy-cm) confirmed a comminuted condylar fracture with associated angulation, impaction, and approximately 5 mm medial translation (Fig. 3). There were no additional mandibular, facial bone, or intracranial injuries. Due to the mechanism and age of the patient, a non-accidental trauma evaluation was initiated. Skeletal survey and laboratory screening were unremarkable. Plastic surgery recommended conservative management with no immediate surgical intervention and advised routine follow-up imaging. The family was counseled on the potential for long‑term sequelae such as temporomandibular joint pain.

### Case 4

**Fig. 4:**
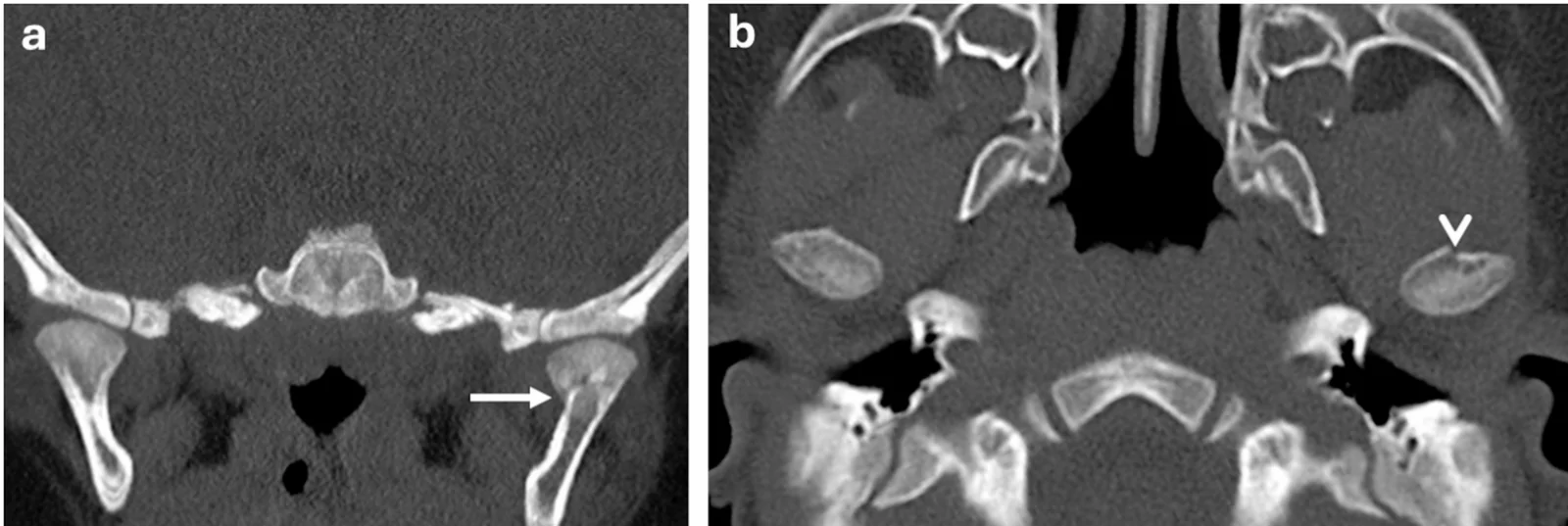
Coronal (**a**) and axial (**b**) bone window CT images from a 4-month-old infant evaluated after falling face down from a 3-foot-high changing table. The images show a nondisplaced intracapsular fracture of the left mandibular condyle (*arrow*); the axial view shows the cortical break (*arrowhead*)

A 4-month-old female infant presented to the emergency department after she rolled off a 3-foot-high changing table and was found face-down on the floor. Over the following hours, her parents observed increased irritability and decreased nursing. Physical examination demonstrated no focal findings. Given concerns for behavioral changes, CT was obtained to evaluate extent of head trauma using low dose protocols that achieved a CTDL vol of 14 mGy and a total DLP of 228.8 mGy-cm. It revealed no acute intracranial abnormalities; however, a non-displaced fracture of the left mandibular condyle with extension to the articular surface was seen. (Fig. 4). She was treated with analgesics and referred to the plastic surgery service for outpatient follow-up. No evaluation for NAT was initiated.

### Case 5

**Fig. 5:**
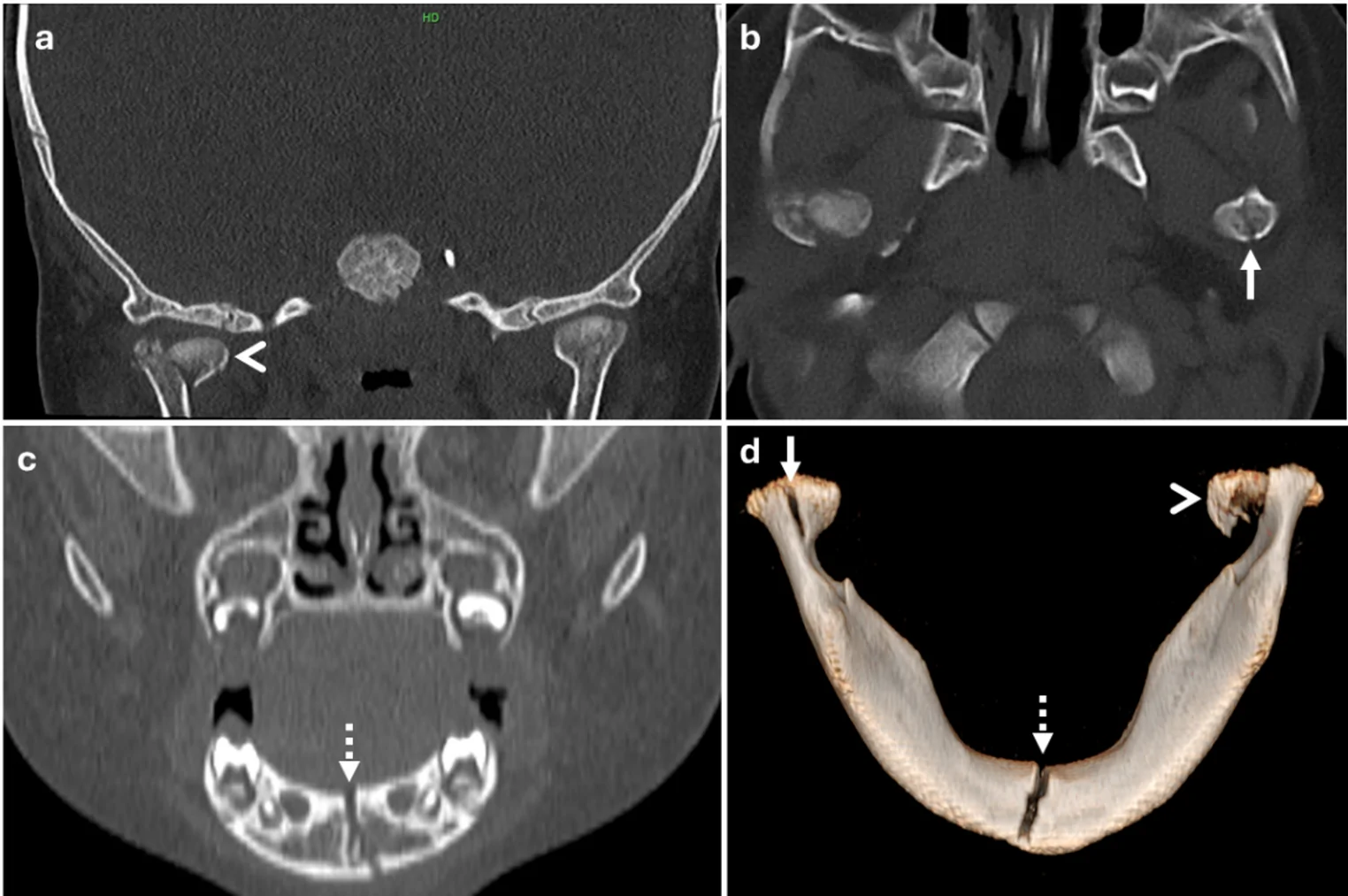
CT scan of a 11‑month‑old girl evaluated for suspected non‑accidental trauma. **a** Coronal and **b** axial images demonstrate a right comminuted and displaced mandibular condylar fracture (*arrowhead*) as well as a left nondisplaced condylar fracture with sclerotic changes (*arrow*). **c** Coronal image of the facial bones reveals a midline mandibular fracture extending superiorly into the alveolar process (*dashed arrow*). **d** Posterior view of a 3D volume‑rendered reconstruction shows all three fractures in a single image

An 11-month-old girl was evaluated for suspected nonaccidental trauma after presenting with facial and abdominal bruising. Initial low dose head CT (CTDL vol – 20.65 mGy and total DLP of 332 mGy-cm) identified nondepressed fractures of the right parietal and occipital bones, and bilateral depressed fractures of the right parietal and occipital bones, and bilateral mandibular condylar fractures (Fig. 5). Maxillofacial CT done with similar low dose protocol, further demonstrated an anterior midline mandibular fracture extending to the left central incisor root, with a comminuted and medially displaced right condylar fracture and a sclerotic left condylar fracture suggestive of a healing process (Fig. 5). A skeletal survey showed an oblique femoral meta-diaphyseal fracture and a right rib fracture. Additional findings included corneal abrasions, and a right thigh burn wound. Her femoral fracture was managed with splint immobilization and analgesia, and the mandibular fractures were treated conservatively as per plastic surgery recommendations. Following a multidisciplinary assessment, the child was placed in foster care.

## Discussion

Mandibular fractures are infrequent in neonates and infants. When mandibular fractures occur in children, they have been shown to have different patterns in different age groups. Younger patients are more susceptible to condylar head and neck fractures, while ramus, and angle fractures are more common in older children [7]. Consistent with existing literature, our findings reflect the characteristic age-related patterns of mandibular condyle fractures. Intracapsular fractures predominated in our cohort of infants, contrasting with higher incidence of condylar neck fractures seen in older children [1].

Diagnosing mandibular condyle fractures in infants and toddlers presents a significant clinical challenge. Because young children are often non-verbal, they cannot communicate subjective symptoms like jaw pain or malocclusion, and physical examinations may be inconclusive as in all our cases [7]. The refusal to feed seen in our third case aligns with the findings of Koti et al., who identified this as the most frequent presenting symptom in their series of young children [8], but this was not a presenting or follow-up feature in the two younger infants. As these injuries often stem from indirect trauma, they can easily be missed, potentially leading to long-term complications such as TMJ pain, facial asymmetry, and malocclusion. Beyond structural changes, they can cause functional disturbances in mastication and speech [1, 2, 5].

The limitations of clinical assessment in toddlers elevates the importance of diagnostic imaging. None of our cases were clinically suspected to have mandibular fractures before imaging. Because of overlapping skull base anatomy, conventional radiography is challenging to interpret, making CT the preferred modality for the detection of condylar fractures [1]. Despite the diagnostic superiority of CT in detecting condylar injuries, the heightened radiosensitivity of infants precludes its use as a universal screening tool [9]. We advocate for a selective imaging strategy and, where CT is indicated, the use of pediatric-specific dose-reduction protocols, as utilized in our study cohort [10, 11]. Notably, the incidental detection of condylar fractures on head CT in our case series emphasizes that radiologists should systematically evaluate the mandibular condyles on all CTs performed for head trauma.

Additionally, recognition of anatomical variants like a bifid condyle is crucial to avoid ‘overcalling’ fractures. A recent systematic review and meta-analysis including 88,625 subjects reported a pooled prevalence of approximately 1% for bifid condyle [12]. The presence of a cortical break, as seen in all our cases, can be used to distinguish an acute fracture from a bifid condyle [13]. One proposed mechanism for the development of bifid mandibular condyles is as a sequel to accidental trauma, with unrecognized condylar fractures healing aberrantly and leading to condylar duplication [12]. Our findings underscore that these fractures can escape clinical detection and, without careful review, may also be overlooked on imaging.

Given the limited mobility of infants, these fractures should also prompt suspicion of NAT as illustrated in Case 5 of our series. In the case series by Koti et al., involving infants and toddlers from two hospitals, most mandibular injuries in children were deemed accidental rather than abusive or indeterminate. Nevertheless, they emphasized the importance of considering NAT, even when a plausible fall history is provided [8]. Similarly, most cases in our series resulted from accidental trauma, but in 4 out of 5 cases, patients underwent NAT protocols including skeletal surveys. In Case 1, suspicion of NAT was secondary to a repeat traumatic event; Cases 2 and 3 underwent NAT evaluation during their initial evaluation. Case 4 was not evaluated for NAT, and Case 5 was confirmed as having NAT.

Conservative management is the predominant and preferred approach for pediatric mandibular condyle fractures, with 72.1% of patients treated nonoperatively in one large series, with excellent outcomes achieved across all age groups [14]. Current consensus recommends that nondisplaced and minimally displaced fractures should be treated expectantly [2, 15, 16], which is consistent with management in all cases in our series. Shi et al. suggest that surgical treatment might be warranted in severely displaced fractures (> 55° angulation) [15].

## Conclusion

Given that infants cannot report symptoms, and physical examination findings may be subtle, radiologists’ identification of mandibular condylar fractures on trauma head CT scans is critical for their diagnosis. Our series focuses on infants, a population with limited independent mobility. The presence of a mandibular condyle fracture coupled with a mechanism of injury that is biomechanically inconsistent with the clinical findings would necessitate workup for NAT. Recognition of these fractures also informs management and counseling regarding possible complications.

## Data Availability

No datasets were generated or analysed during the current study.
